# New Appliances and Things Medical

**Published:** 1901-10-12

**Authors:** 


### NEW APPLIANCES AND THINGS MEDICAL.

[We shall be glad to receive, at our Office, 28 & 29 Southampton Street, Strand, London, W.C., from the manufacturers, specimens of all new preparations and appliances which may be brought out from time to time.]

### JACKSON'S CAMPHORATED WAX POLISH.

(T. S. Jackson and Sons, 168 Old Kent Road, London, S.E.)

We have examined the above camphorated wax polish, and find it a carefully-prepared paste of pure manufacture, exceedingly valuable for cleaning leather goods, such as brown boots, and for polishing furniture and parquet flooring. It is only necessary to apply a little of the wax with a piece of flannel, and then to rub well with a soft cloth. One of the chief advantages of Jackson's polish is that the best results are obtained by using a small amount of wax, and rubbing till all the excess is removed; this procedure means absolute cleanliness, and the removal of extraneous dirt and *débris*. This polish does not hide and disguise dirt, it removes it, a very valuable property from a sanitary and hygienic aspect.

### WHOOPING-COUGH LOTION.

(G. Hayward, 25 Exmouth Road, Southsea.)

We have received a sample of the above preparation. The descriptive title of the preparation is hardly complete. A lotion implies practically any fluid substance for outward application—a generic term which comprises embrocations, fomentations, liniments, collyria, etc. No directions for use have been supplied with the sample forwarded us, so that we hardly understand the method by which it should be applied. From the oleaginous nature of the fluid we infer it is intended to be used as an embrocation for rubbing into the chest, back and front of patients suffering from whooping-cough, and when used in this manner we can conceive that it may have a favourable influence on the course of the disease by bringing the skin into action—always a desirable consummation in diseases of this nature. We cannot, however, detect the presence of any substance contained in the lotion which is likely to have a specific effect on the disease.

### REFINED BEEF SUET.

(Hugon and Company, Ltd., Pendleton, Manchester.)

In a former issue we have referred in these columns to that excellent form of beef suet known as Hugon's. The great advantage of this suet is that it is very largely freed from the water which invariably occurs in fats in their fresh and natural state. This presence of water and certain other substances is the reason why ordinary suet will not keep for any length of time, whereas Hugon's, being practically a pure fat, will keep almost indefinitely; it can be kept in store, and therefore be ever present at hand for immediate use. In this respect it is obviously an economical preparation. Further than this, it can be so easily shredded with a knife that there is a great saving in labour as compared with the usual method of chopping which is necessary when ordinary suet is employed. It is eminently a cleanly and hygienic preparation for culinary use.

